# First isolation and analysis of caesium-bearing microparticles from marine samples in the Pacific coastal area near Fukushima Prefecture

**DOI:** 10.1038/s41598-021-85085-w

**Published:** 2021-03-11

**Authors:** Hikaru Miura, Takashi Ishimaru, Yukari Ito, Yuichi Kurihara, Shigeyoshi Otosaka, Aya Sakaguchi, Kazuhiro Misumi, Daisuke Tsumune, Atsushi Kubo, Shogo Higaki, Jota Kanda, Yoshio Takahashi

**Affiliations:** 1 Atmospheric and Marine Environmental Sector, Environmental Science Research Laboratory, Central Research Institute of Electric Power Industry, 1646 Abiko, Abiko, Chiba 270-1194 Japan; 2 Department of Ocean Sciences, Graduate Faculty of Marine Science, Tokyo University of Marine Science and Technology, 4-5-7 Konan, Minato-ku, Tokyo, 108-8477 Japan; 3 Ningyo-Toge Environmental Engineering Centre, Japan Atomic Energy Agency, 1550 Kamisaibara, Kagamino-cho, Tomata-gun, Okayama, 708-0698 Japan; 4 Atmosphere and Ocean Research Institute, The University of Tokyo, Kashiwanoha 5-1-5, Kashiwa, Chiba 277-8564 Japan; 5 Centre for Research in Isotopes and Environmental Dynamics, University of Tsukuba, 1-1-1 Tennodai, Tsukuba, Ibaraki 305-8577 Japan; 6 Department of Geosciences, Faculty of Science College of Science, Academic Institute, Shizuoka University, 836 Ohya, Suruga-ku, Shizuoka, 422-8529 Japan; 7 Isotope Science Centre, The University of Tokyo, 2-11-16 Yayoi, Bunkyo-ku, Tokyo, 113-0032 Japan; 8 Department of Earth and Planetary Science, Graduate School of Science, The University of Tokyo, 7-3-1 Hongo, Bunkyo-ku, Tokyo, 113-0033 Japan

**Keywords:** Environmental sciences, Environmental chemistry, Marine chemistry

## Abstract

A part of the radiocaesium from the Fukushima Daiichi Nuclear Power Plant (FDNPP) accident was emitted as glassy, water-resistant caesium-bearing microparticles (CsMPs). Here, we isolated and investigated seven CsMPs from marine particulate matter and sediment. From the elemental composition, the ^134^Cs/^137^Cs activity ratio, and the ^137^Cs activity per unit volume results, we inferred that the five CsMPs collected from particulate matter were emitted from Unit 2 of the FDNPP, whereas the two CsMPs collected from marine sediment were possibly emitted from Unit 3, as suggested by (i) the presence of calcium and absence of zinc and (ii) the direction of the atmospheric plume during the radionuclide emission event from Unit 3. The presence of CsMPs can cause overestimation of the solid–water distribution coefficient of Cs in marine sediments and particulate matter and a high apparent radiocaesium concentration factor for marine biota. CsMPs emitted from Unit 2, which were collected from the estuary of a river that flowed through a highly contaminated area, may have been deposited on land and then transported by the river. By contrast, CsMPs emitted from Unit 3 were possibly transported eastward by the wind and deposited directly onto the ocean surface.

## Introduction

Large amounts of radionuclides were emitted into the environment by the Fukushima Daiichi Nuclear Power Plant (FDNPP) accident. Among the many radionuclides emitted, radioactive caesium (RCs) has been intensively investigated because of the large amount of emission and its relatively long half-life (*T*_1/2_ of ^137^Cs = 30 years). Previous studies have shown that 3–6 PBq of ^137^Cs was deposited on the land, 12–15 PBq was deposited from the atmosphere onto the Pacific Ocean surface, and 3.6 ± 0.7 PBq was released directly from the accident site into the ocean^1,2^. RCs concentrations in surface seawater in the coastal area near the FDNPP decreased rapidly during the several years following the accident, partly because of the physical decay of RCs, but mostly because of dispersion by seawater circulation^1,3,4^. However, particulate RCs concentrations in coastal sediments showed large variations^5^ and decreased more slowly than the dissolved RCs concentrations^6^. We hypothesized that the large variations and slow decrease of particulate RCs concentrations might be due to the presence of water-resistant Cs-bearing microparticles (CsMPs); this possibility was also suggested by Kusakabe et al*.*^7^ and Otosaka^8^.

Adachi et al*.*^9^ first reported CsMPs from the FDNPP accident on filters used to collect aerosols in Tsukuba, Ibaraki Prefecture (this type of CsMPs are known as Type-A particles following Igarashi et al*.*^10^). Subsequent studies have shown that the matrix of Type-A particles is silicon dioxide (SiO_2_) glass, and Cs, iron (Fe), zinc (Zn), and other various elements are included in the matrix^11–14^. Type-A particles are ~ 0.1–10 µm in diameter, and their ^137^Cs activity is ~ 10^–2^ to 10^2^ Bq/particle^10,15^. Uranium (U) and Cs isotopic ratios (U isotopes, ^235^U and ^238^U; Cs isotopes, ^133^Cs, ^134^Cs, ^135^Cs, and ^137^Cs)^16^, and ^134^Cs/^137^Cs activity ratios^17^ of Type-A particles are consistent with those of Unit 2 or 3 (Unit 2/3 hereinafter) of the FDNPP calculated theoretically based on the nuclear fuel burnup^18^. Another type of CsMPs, Type-B particles^10^, emitted from Unit 1 of the FDNPP was collected at the vicinity of the FDNPP^19^. Type-B particles are also composed mainly of SiO_2_, but they are variable in shape and range in diameter from 50 to 400 μm. Their ^137^Cs activity is 10^1^–10^4^ Bq/particle, and they include refractory elements such as calcium (Ca)^17,20^.

Type-A particles were discovered from suspended particles in the downstream of the Kuchibuto River in Fukushima Prefecture^21^. Previous studies did not succeed in isolating CsMPs from suspended particles in seawater and marine sediments, although they reported highly radioactive spots on autoradiographic images of marine samples^22,23^. The presence of CsMPs in river water and the radioactive spots on autoradiographic survey images of the marine samples suggest that CsMPs, which deposited on the land, eventually flow into the ocean via rivers. To confirm this possibility, the particles must be isolated and directly observed, for example by conducting a scanning electron microscopy (SEM) with energy-dispersive spectrometer (EDS) analysis, because CsMPs and RCs-enriched clay minerals are hard to distinguish on autoradiographic images^15^. Despite the importance of CsMPs in evaluating the behaviour of RCs in the ocean and its influence on marine biota, to the best of our knowledge CsMPs have not yet been isolated from marine samples. In this study, we efficiently isolated CsMPs from marine samples collected in coastal areas of the Pacific Ocean by the wet separation method^16,17,21^. After isolating CsMPs from the marine samples, we conducted an SEM–EDS analysis of the isolated CsMPs and measured their radioactivity with a high-purity germanium semiconductor (HPGe) detector. We also measured their size, elemental composition, ^137^Cs activity, and ^134^Cs/^137^Cs activity ratio, and we calculated the ratio of ^137^Cs in CsMPs to the total ^137^Cs in the sample. That ratio affects evaluations of (i) the solid–water distribution coefficient (*K*_d_) value between seawater and particulate matter in the water column or marine sediments, and (ii) the RCs concentration factor (CF) for marine organisms. Moreover, we discussed the sources of CsMPs in the marine samples and their migration behaviour in the ocean.

## Results and discussion

**Figure 1:**
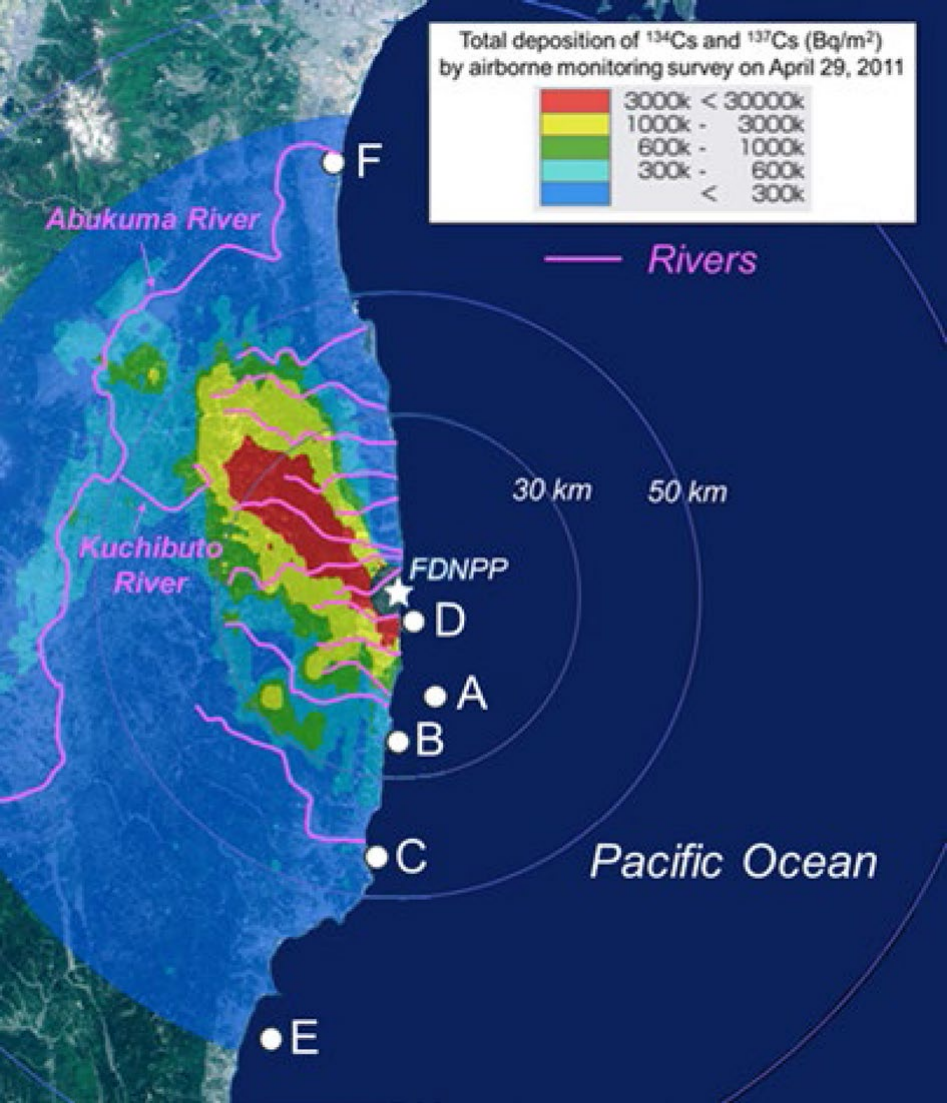
Sampling points A–F and rivers near the FDNPP, shown on a Cs inventory map from MEXT^40^.

We isolated seven radioactive particles (RPs) from the marine samples collected at points A–F (Fig. 1). We refer to the five RPs isolated from samples of particulate matter (suspended particles, sinking particles, and zooplankton) as PM particles; those from points A, B, C, D, and F are referred to as PM-A, -B, -C, -D, and -F, respectively. We refer to the two RPs isolated from the marine sediment sample collected at point E as MS particles (MS-E1 and -E2).

### RCs activity in RPs isolated from bulk marine samples

The analytical data for the bulk samples and isolated RPs are summarized in Table 1 (all RCs data have been decay-corrected to 11 March 2011). The ^134^Cs and ^137^Cs activities of the PM particles were 0.250–1.83 Bq and 0.229–1.66 Bq, respectively, whereas the ^134^Cs and ^137^Cs activities of the MS particles were 20.4–42.0 Bq and 18.6–38.6 Bq, respectively. These differences are attributable to the difference in the particle sizes (MS particles > PM particles). The ^134^Cs/^137^Cs activity ratio of all RPs was approximately 1 (average = 1.09); therefore, the RPs isolated from the marine samples were derived from the FDNPP accident^18^. In addition, the ^134^Cs/^137^Cs activity ratios of the RPs (~ 1.07 to 1.11; Table 1) indicated that they were emitted from Unit 2/3 of the FDNPP because the ratios for RPs were around 1.05 (Unit 3) and 1.08 (Unit 2) calculated by Nishihara et al*.*^18^.

**Table 1:** Data for the bulk samples and the isolated radioactive particles (RPs).

Sampling point	Sampling date	Distance from the FDNPP (km)	Bulk				Particle					A (RPs)/A (Bulk) (%)
			Sample name	Total ^137^Cs activity (Bq)	^137^Cs activity concentration (Bq/kg-dry)^b, c^	Filtered water volume (L)	Name	Size (μm)	^137^Cs activity (Bq) ^b,c^	^137^Cs activity per unit volume (Bq/mm^3^)	^134^Cs/^137^Cs activity ratio^b,c^	
	yyyy/mm/dd			A (Bulk)					A (RPs)			R (RPs)
A	2015/07/29	18	P-43-I01	8.1	26,852 ± 385	2583	PM-A	2.5	1.57 ± 0.01	1.8 × 10^8^	1.09 ± 0.10	19
				1.2	3230 ± 103		–	–	–	–	–	–
B	2013/12/16	24	Hisanohama	13	7747 ± 46	630	PM-B	1.5	0.229 ± 0.003	1.2 × 10^8^	1.09 ± 0.10	1.8
C	2011/07/02	43	UT06	2.2	16,590 ± 354	119,300	PM-C	1.1, 0.7	1.66 ± 0.01	1.8 × 10^9^	1.10 ± 0.02	77
D^a^	2014/10/17	5	GST#1	6.1	877 ± 42	–	–	–	0.339	–	–	5.6
	2014/10/26		GST#2	7.9	669 ± 30		–	–	0	–	–	0
	2014/11/04		GST#3	10	1424 ± 69		–	–	0.338	–	–	3.3
	2014/11/13		GST#4	84	1175 ± 43		–	–	0.981	–	–	1.2
	2014/11/22		GST#5	39	1539 ± 72		PM-D	1.7	1.36 ± 0.01	5.3 × 10^8^	1.11 ± 0.10	3.5
	2014/12/01		GST#6	24	753 ± 36		–	–	0.546	–	–	2.3
	2014/12/10		GST#7	35	1278 ± 63		–	–	1.14	–	–	3.3
E	2011/07/20	75	4UB06	61.4	8709 ± 47	–	MS-E1	~ 10	38.6 ± 0.1	1.1 × 10^9^	1.09 ± 0.05	93
							MS-E2	~ 15	18.6 ± 0.1	7.3 × 10^8^	1.10 ± 0.07	
F	2012/11/30	70	Watari	2.8	1360 ± 80	60	PM-F	1.8	0.687 ± 0.009	2.2 × 10^8^	1.07 ± 0.09	25

^a^At sampling point D, a RP was isolated only from GST#5; other A (RPs) values were calculated from the photo stimulated luminescence of the imaging plate images.

^b^Values are decay-corrected to 11 March 2011.

^c^The 1σ error is based on the counting statistics.

### Characterisation of the isolated RPs

**Figure 2:**
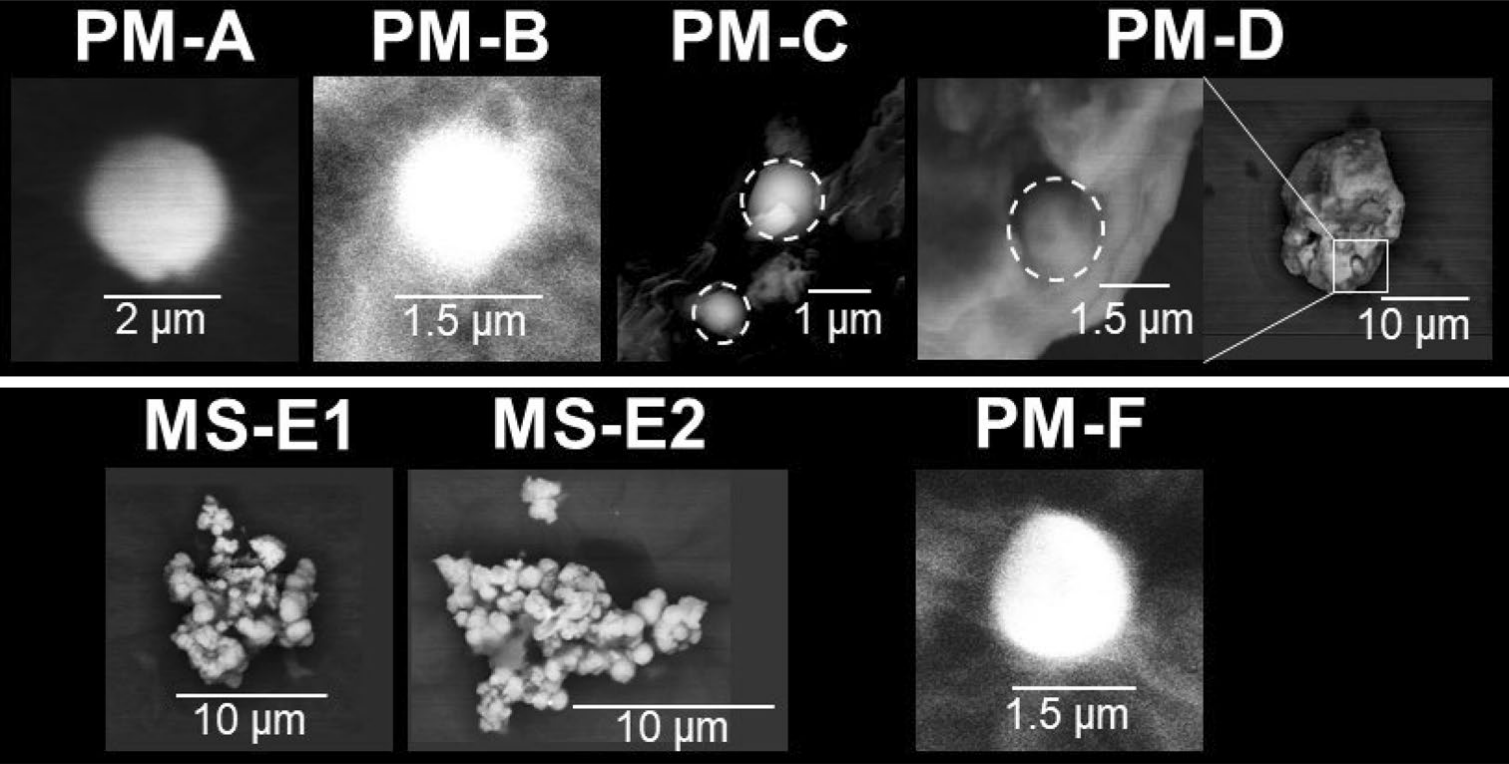
SEM images of the seven radioactive particles (RPs) isolated from marine samples. PM and MS indicate RPs isolated from particulate matter and marine sediment, respectively. RPs were placed on Kapton tape for the SEM–EDS analyses. Cs was detected only in areas within the white dashed circles of each large particles incorporating PM-C and -D.

**Figure 3:**
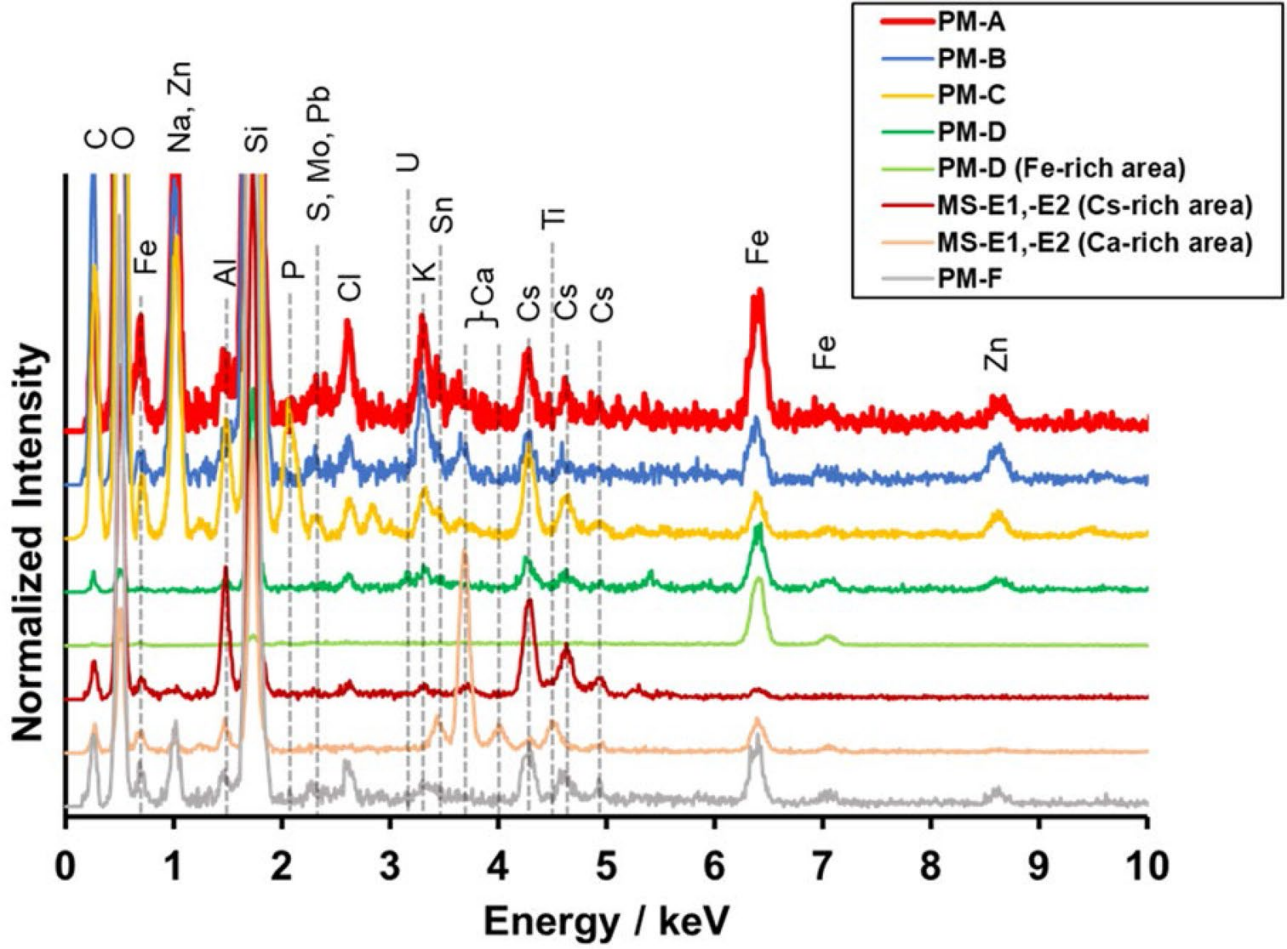
EDS spectra of the seven RPs. The carbon was from the carbon coating done during sample preparation.

PM-A, -B, and -F were similar to terrestrial Type-A particles^17^ with regard to their size and shape (Fig. 2), elemental composition (Fig. 3, Table 2), and RCs activity (Table 1). PM-C and -D were attached on a fiber-reinforced plastic particle (~ 2 mm) and Fe-rich particle (~ 20 µm), respectively. Although Cs was detected only in an area with a radius of about 1 µm on these larger particles (within the white dashed circles in Fig. 2), Si, chlorine, potassium, Fe, and Zn peaks were detected on the EDS spectra of these areas; thus, they were compositionally similar to Type-A particles from terrestrial samples^17^. It was necessary to obtain elemental maps of PM-D by EDS (Fig. S1) because the area where Cs was detected could not be distinguished on backscattered electron images. The EDS spectrum of PM-D showed a weak but clear U peak (Fig. 3). Abe et al*.*^11^ showed by a synchrotron radiation analysis that Type-A particles from a terrestrial sample contained U. The aluminium (Al) detected in some PM particles is probably attributable to secondary adhesion of Al to the particles surface because previous studies scarcely detected Al inside CsMPs^12–14^.

**Table 2:** Comparison of radioactive particle (RP) types.

	Previous studies			This study		
	Type-A particles^a^	Type-B particles^a,b^	SP^c^	MS-E1 and -E2		PM-A, -B, -C, -D, and -F
				Cs-rich area	Ca-rich area	
Origin estimated from the activity ratio (^134^Cs/^137^Cs) (Unit 2/3, (^134^Cs/^137^Cs) > 1; Unit 1, (^134^Cs/^137^Cs) < 1)	Unit 2/3	Unit 1	Unit 2/3	Unit 2/3		Unit 2/3
Particle size (μm)	~ 0.1–10	50–400	120	~ 10		~ 1–2
^137^Cs activity per unit volume (Bq mm^-3^)	~ 10^8^–10^9^	~ 10^4^–10^6^	~ 10^4^–10^5^	~ 10^8^–10^9^		~ 10^8^–10^9^
**Major elements (except Si and O**^**d**^**)**						
Mg	×	○	○	×	×	×
Al	×	○	○	○	○	△
Cl	○	×	×	△	×	○
K	○	○	○	△	×	○
Ca	×	○	×	△	○	×
Ti	×	○	○	×	○	×
Fe	○	○	○	△	○	○
Zn	○	○	×	×	×	○
Sn	○	×	×	×	○	○
Cs	○	×	×	○	△	○

^a^Data from Miura et al*.*^17^.

^b^Data from Ono et al*.*^19^.

^c^CsMP reported by Zhang et al*.*^24^ (particle name: SP).

^d^○, detected; △, detected in only some particles or only a slight amount detected; ×, not detected.

**Figure 4:**
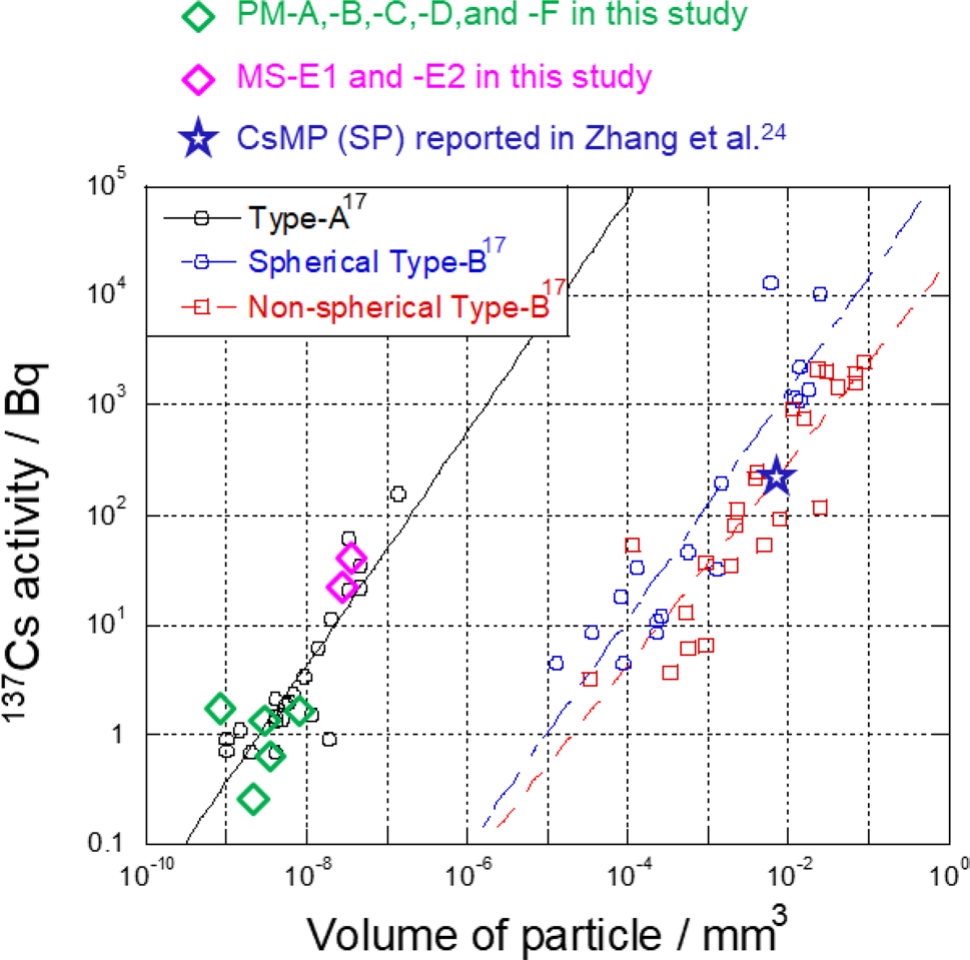
Relationships between ^137^Cs activity and particle volume (^137^Cs activity per unit volume) of Type-A and Type-B particles, reported by Miura et al*.*^17^, compared with that of the marine RPs in this study. ^137^Cs activity per volume of RPs from marine samples is similar to that of terrestrial Type-A particles. The relationship in sample SP reported by Zhang et al*.*^24^ is also plotted.

The volume of each RP was calculated from its apparent diameter and compared with CsMP volume data reported by Miura et al*.*^17^ (Fig. 4); the diameters of the white dashed circles on the SEM images (Fig. 2) were used to calculate the volumes of PM-C and -D. In terrestrial samples, the ^137^Cs activity per unit volume of Type-A particles is ~ 10,000 times higher than that of Type-B particles emitted from Unit 1 of the FDNPP^17^ (Fig. 4). The RPs isolated from the marine samples in this study plotted approximately along the line corresponding to the relationship in Type-A particles from terrestrial samples. Thus, the PM particles in this study were classified as Type-A particles on the basis of their elemental composition (Table 2), ^134^Cs/^137^Cs activity ratio (Table 1), and ^137^Cs activity per unit volume (Table 2, Fig. 4).

The MS particles were found to be aggregates of Type-A particles. MS-E1 and -E2 were ~ 10 µm and ~ 15 µm in size, respectively. To calculate the volumes (Fig. 4), we approximated their volume by summing the volumes of the spheres composing each aggregate (Fig. S2). Because the two MS particles were not wholly visible in the SEM images, MS particle volume was underestimated. The ^134^Cs/^137^Cs activity ratio (Table 1) and ^137^Cs activity per unit volume of the MS particles were similar to those of Type-A particles. However, although the Type-A particles reported so far contain Zn^10^, no Zn was detected in the MS particles (Fig. 3, Table 2). Moreover, the MS particles included a Ca-rich area that overlapped the Cs-rich area (Figs. 3, S3, and S4). This characteristic is not found in the previously reported CsMPs (i.e., Type-A and Type-B particles)^17,19^. Zhang et al*.*^24^ considered a RP that they called SP, which had a ^134^Cs/^137^Cs activity ratio of more than 1 and lacked Zn, to be a new type of CsMPs (Table 2). However, SP differed from the MS particles with regard to ^137^Cs activity per unit volume (SP < MS-E1 and -E2; Fig. 4, Table 2) and elemental composition (e.g., Ca; Table 2). Therefore, the MS particles represent a new type of RP that is different from the previously reported CsMPs^17,19^ and SP^24^.

### Influence of CsMPs on *K*_d_ and CF values

We calculated the ratio of ^137^Cs activity in RPs to the total ^137^Cs in the bulk sample (R(RPs)) (Table 1). In the bulk samples collected from points A, B, C, E, and F, no radioactive spots were found by autoradiography with imaging plate (IP) apart from the isolated RPs. By contrast, most of the time-series samples collected at point D (GST#1–7) had radioactive spots, although a RP was isolated from only GST#5 (Table 1). In the current study, we assumed that radioactive spots with ^137^Cs activity of > 0.1 Bq on the IP image were CsMPs and calculated R(RPs) of GST#1–7 by using the photo-stimulated luminescence (PSL) values of the IP images (Table 1)^21^. The R(RPs) values of the zooplankton sample from point C and the sediment sample from point E were high, possibly because the concentration of non-RP ^137^Cs present as adsorbed species on the bulk samples was low. Both of these samples were collected in 2011, but the mesh size of the plankton net used to collect the sample from point C was 330 µm, and the < 2 mm fraction of sediment was recovered from the sample at point E by sieving. Consequently, the total surface area of the samples onto which Cs could be adsorbed was small and the ^137^Cs concentration of the bulk samples was low. In addition, the higher ^137^Cs activity of MS particles (~ 57 Bq), compared with the other RPs, caused R(RPs) of the sample from point E to be relatively high.

The large variation of RCs activity in marine sediments reported by Kusakabe et al*.*^5^ might be explained by the presence of CsMPs in the ocean. In a sample that included CsMPs, the *K*_d_ value of Cs would be overestimated because the Cs in CsMPs would be considered to be adsorbed on clay minerals. Similarly, the CF value would be overestimated because the Cs would be considered to have been absorbed by plankton. In actuality, however, Cs in CsMPs does not affect *K*_d_ or CF values because of the water-resistant character of CsMPs. CsMPs in rivers have also led to variation of Cs *K*_d_ values^21^. When the amount of Cs adsorbed to clay minerals (absorbed by plankton) is assessed on the basis of its *K*_d_ (CF) value, the Cs activity of CsMPs should be excluded. In the current study, radioactive spots with > 0.1 Bq of ^137^Cs activity were assumed to be CsMPs. However, Okumura et al*.*^15^ have reported that some CsMPs have ^137^Cs activity of less than 0.1 Bq, and weathered biotite may have ^137^Cs activity of more than 0.1 Bq; thus, distinguishing between CsMPs and weathered biotite by using IP images is difficult. In the future, a non-destructive method should be developed to allow them to be distinguished.

### Source of CsMPs in marine samples

A key question of this study was where did the CsMPs in the marine samples come from? Widespread transport of CsMPs has been linked to flows of radioactive plumes in the atmosphere^9^. Nine major RCs-bearing plumes (P1–P9) from the FDNPP have been reported, based on atmospheric diffusion model calculations^25^ and the measurement of Cs activity concentrations in the atmosphere around eastern Japan^26^. These plumes were released between 12 March 2011 (Plume 1: P1) and 21 March 2011 (Plume 9: P9)^25^. In this study, we used the plume numbers defined by Nakajima et al*.*^25^ and Tsuruta et al*.*^26^.

**Figure 5:**
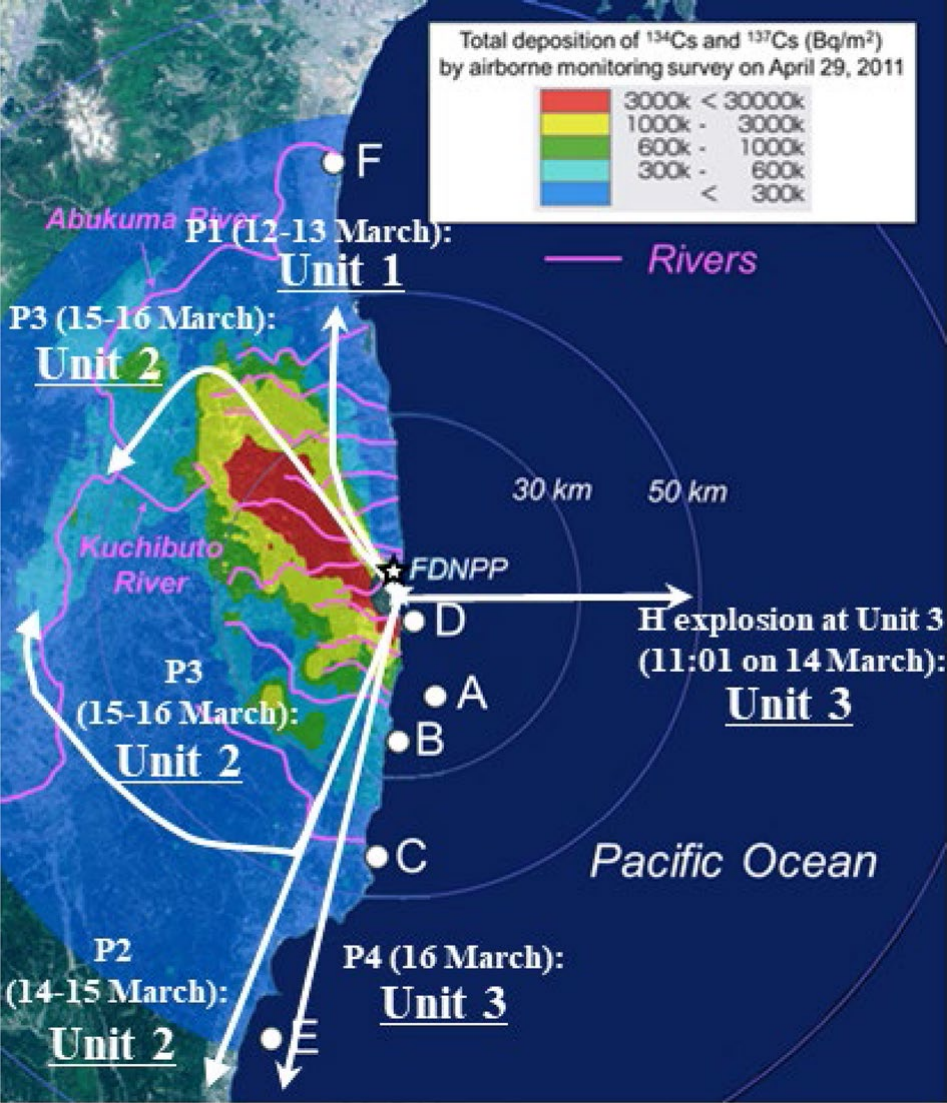
Transport routes of plumes possibly including CsMPs. The date of plume was cited from Tsuruta et al.^26^, and the transport route was cited from Nakajima et al.^25^.

P1 (emitted 12–13 March 2011), which included Type-B particles, was emitted from Unit 1 of the FDNPP at 15:36 JST on 12 March 2011 as a result of a hydrogen (H) explosion, and it flowed north-westward^27,28^ (Fig. 5). Type-B particles derived from this explosion were found to be distributed in a very narrow area north-northwest of the FDNPP^17,19,20^.

According to the International Research Institute for Nuclear Decommissioning (IRID)^29^, the Zn in Type-A particles was derived from coating materials in the FDNPP suppression chamber (S/C) because the water source for reactor Unit 2 isolation cooling was switched from a condensate storage tank to the S/C. Thus, the IRID^29^ concluded that Type-A particles were from Unit 2 of the FDNPP. In support of the IRID conclusion^29^, Kurihara et al*.*^16^ inferred that Unit 2 was the emission source of Type-A particles (particularly spherical ones) on the basis of (i) U and Cs isotope ratios, (ii) the sequence of events during the accident^30,31^, and (iii) reported temporal variations of polluted air^25–28,32^. Type-A particles were collected on an air filter on 14–15 March 2011 in Tsukuba^9^; therefore, P2 (emitted from the night of 14 March until the morning of 15 March 2011) contained Type-A particles. P2 flowed southwest toward the Tokyo Metropolitan Area (TMA). Ikehara et al.^33^ showed by an autoradiography analysis of IP of contaminated soil samples that both P2 and P3 (emitted on the afternoon of 15 March 2011) contained Type-A particles. P3 flowed first toward the central part of Fukushima Prefecture, and later northward and northwestward. Therefore, in this study we considered that both P2 and P3 included Type-A particles emitted from Unit 2 (Fig. 5).

Considering that wet venting reduced particulate emissions, CsMPs may have been released from Unit 3 during two events. The first was a H explosion that occurred at 11:01 JST on 14 March 2011, and the other occurred on the morning of 16 March 2011 and produced P4. The decreasing dry well pressure^31^ and white smoke from the Unit 3 building^32^ observed on 16 March indicated that RCs in P4 were derived mainly from Unit 3^27^. At the time of the H explosion, the wind was blowing towards the east. P4 flowed towards the south-southwest direction and was transported to offshore of the TMA^25^ (Fig. 5).

P5 (18 March 2011) and P6 (19 March 2011) were transported northward from the FDNPP; then, after reaching the northern coastal area about 50 km from the plant, they flowed northeast. It is not clear whether P5 and P6 contained CsMPs, but their transport pathways did not overlap any of the sampling points of this study. P7 (20 March 2011) flowed first toward the Pacific Ocean and subsequently the northern part of the TMA. P8 (20–21 March 2011) was transported to the northwest about 50 km from the plant and then gradually moved southward. P9 (21 March 2011) flowed southeast and spread over a wide area of the TMA. Radioactive materials were released continuously during the origin periods of the P7–P9^25^, and no CsMPs were collected on air filters during 20–21 March in Tsukuba^9^; thus, P7–P9 probably contained no CsMPs. Therefore, we focused on P1–P4 and suggested three possible sources of Type-A particles in the marine samples: rivers, direct atmospheric deposition, and direct release to the ocean.

#### (1) River transport

P2 and P3 deposited Type-A particles mainly on land areas (Fig. 5). In the five years after the FDNPP accident, 12 TBq of ^137^Cs was transported to the ocean by the Abukuma River and almost all of the RCs (96.5%) were transported in particulate form^34^. Miura et al*.*^21^ has reported Type-A particles in suspended particles from the Kuchibuto River (Fig. 1), a tributary of the Abukuma River. PM-F, a Type-A particle, was found in the sample collected at point F from the Abukuma River estuary. Several other rivers also flow through highly contaminated areas (Fig. 1), and Kubo et al*.*^22^ collected suspended particles with highly radioactive spots from the estuary of the Kuma River (4 km to the south of the FDNPP). These findings indicate that Type-A particles deposited on land after the accident subsequently entered various rivers as a result of surface soil erosion and were transported to the ocean.

#### (2) Direct atmospheric deposition

Few Type-A and Type-B particles were deposited from atmospheric plumes directly onto the ocean surface (Fig. 5). Okumura et al*.*^35^ have suggested on the basis of a dissolution experiment that Type-A particles (1 µm) might dissolve completely in seawater within about 10 years because of the high ionic strength of seawater. Therefore, Type-A particles directly deposited in the ocean might already have dissolved. CsMPs emitted from Unit 3, however, might have been directly deposited on the ocean because at the time of the H explosion, the wind was blowing toward the ocean, and the P4 pathway was also partly over the ocean (Fig. 5).

#### (3) Direct release to the ocean

Direct release of highly radioactive liquid wastes from the FDNPP to the ocean was also a possible source, which was discussed by Tsumune et al*.*^2^. However, whether particulate RCs was released directly from the FDNPP to the ocean remains unknown. Therefore, we cannnot evaluate whether direct release was a source of CsMPs. We therefore inferred that the PM particles in this study were Type-A particles that were deposited on land from P2 and P3, transported to rivers by surface soil erosion and runoff, and then transported via the rivers to the ocean.

In contrast, the MS particles were possibly derived from P4, because sampling point E is along the transport pathway of P4. In addition, the characteristics of the MS particles differed from those of previously reported CsMPs^17^. Furthermore, the molten core concrete interaction was more limited in Unit 2 than in Unit 3^29^, which supported that the MS particles were emitted from Unit 3 because of containing Ca probably derived from concrete. These results and reports suggest that the MS particles were emitted from Unit 3, directly deposited onto the ocean surface, and possibly transported by ocean currents to point E. By contrast, Zhang et al*.*^24^ suggested that SP, which they isolated from a terrestrial soil sample collected from Okuma Town (3 km south–southwest of the FDNPP), was emitted from Unit 3 by H explosion at 11:01 JST on 14 March 2011. They suggested that the force of the explosion transported SP against the wind (Fig. 5) and deposited it on the land because of its large size and high density. We consider it likely that the MS particles in this study and SP came from Unit 3, but more particles need to be analysed to confirm this inference.

### Migration of Type-A particles in the ocean

To understand the migration of Type-A particles in the ocean, we conducted the following calculation to estimate the horizontal transport distance of Type-A particles in the ocean until their deposition on the seafloor.

**Figure 6:**
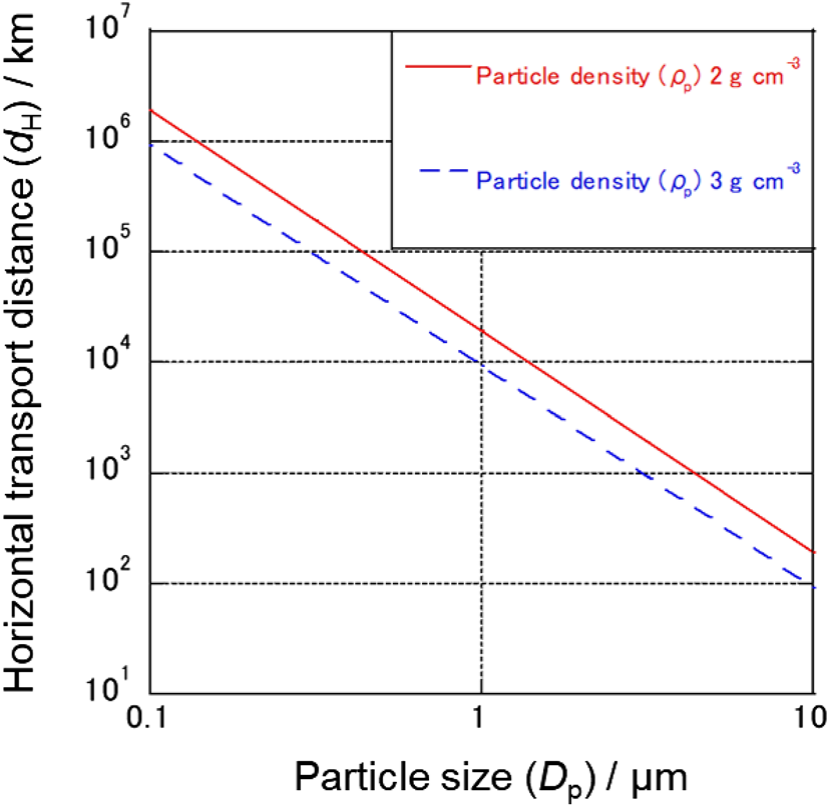
Relationship between horizontal transport distance and particle size by calculation.

The reported diameter of Type-A particles (*D*_p_) ranges from ~ 0.1 to 10 µm, and their density (*ρ*_p_) is assumed to be around 2.5 g cm^–3^, which is the density of soda-lime glass. Thus, in the calculation, we used 2.0–3.0 g cm^–3^ as the range of *ρ*_p_. The density (*ρ*_f_) and viscosity (η) of seawater are 1.024 g cm^–3^ and 1.01 × 10^–2^ g cm^–1^ s^–1^, respectively^36,37^. In accordance with Stokes’ Law, the sinking velocity (*v*_s_) of Type-A particles with *D*_p_ from 0.1 µm to 10 µm can be calculated as follows:

$$v_{{\text{s}}} = \frac{{D_{{\text{p }}}^{2} (\rho_{{\text{p}}} - \rho_{{\text{f}}} ){\text{g}}}}{18\eta }$$

where g is gravitational acceleration. Then, the horizontal transport distance of Type-A particles until they are deposited on the seafloor (*d*_H_) can be calculated with the following equation:

$$d_{{\text{H}}} = d_{{\text{w}}} v_{{\text{H}}} /v_{{\text{s}}}$$

where *d*_w_ and *v*_H_ are water depth and horizontal flow velocity, respectively. If *d*_w_ = 100 m and *v*_H_ = 0.1 m s^–1^ (the order of *v*_H_ was estimated from Tsumune et al*.*^38^), the *d*_H_ of Type-A particles with *D*_p_ of 1 µm would be ~ 10,000 km (Fig. 6). Such particles would likely not be found in coastal sediments because they would be transported further offshore. By contrast, *d*_H_ of Type-A particles with *D*_p_ of 10 µm would be ~ 100 km, and such particles might therefore be found in coastal sediments.

The result of this calculation is consistent with the characteristics of the Type-A particles collected in the current study. PM-A, -B, and -F (diameter ~ 1 µm) were collected from suspended particles, whereas PM-D, MS-E1, and MS-E2 (diameter > 10 µm) were collected from sinking particles and sediment. Moreover, it is important to note that PM-D was attached to an Fe-rich particle, the density of which was likely high, whereas the matrix of PM-C, which was isolated from the plankton net sample, was a low-density plastic (mostly < 1.5 g cm^–3^)^39^.

PM-A and -B, which were collected in July 2015 and December 2013, respectively, from suspended particles in the coastal area, were Type-A particles with a diameter of ~ 1 µm. Because their shape indicated that they had not been altered by dissolution^35^, these CsMPs were likely collected relatively soon after their transport via rivers to the ocean.

## Materials and methods

### Bulk sample collection and measurement of ^134^Cs and ^137^Cs in the samples

Sampling points A–F and rivers near the FDNPP are shown in Fig. 1 on a Cs inventory map from MEXT^40^. Bulk samples were collected by several methods as described below; sampling information is summarized in Table S1.

#### (1) Suspended particles collected by water filtration using large-volume pumps (points A and B)

Large-volume pumps (WTS-LV, McLane Research Laboratories, Inc., Falmouth, MA) were used to collect suspended particles in July 2015 (point A) and December 2013 (point B) by the T/S *Oshoro-maru* of Hokkaido University and the fishing vessel *Koumei-maru*, respectively. The seawater was filtered through a PVDF membrane filter (Durapore, diameter 142 mm, pore size 0.45 μm, Merck Ltd., Tokyo, Japan). Then, the samples of suspended matter were removed from the filters with a plastic spatula, dried at 60 °C, and transferred to Teflon tubes. RCs concentrations of the suspended matter samples were measured by using a gamma-ray spectrometer with a well-type Ge detector (Canberra EGPC 250-P21, Meriden, CT). Samples were prepared for this measurement by the method of Kubo et al*.*^22^.

#### (2) Zooplankton collected by plankton net (point C)

A plankton net with a 330 µm mesh was used to collect zooplankton at point C in July 2011 by the T/V *Umitaka-maru* of the Tokyo University of Marine Science and Technology. The sample was dried at 60 °C and transferred to Teflon tubes. The RCs concentration of the plankton net sample was measured by using a gamma-ray spectrometer with a well-type Ge detector, as described above for the suspended particle samples.

#### (3) Sinking particles collected in a sediment trap (point D)

Sinking particles were collected by a time-series sediment trap (SMC7S-500ex, NiGK Corp., Japan) at point D between October and December 2014. The sinking particle samples were dried at 60 °C and transferred to Teflon tubes. RCs concentrations in the sinking particle samples were also measured by using a gamma-ray spectrometer with a well-type Ge detector.

#### (4) Marine sediment collected by a grab sampler (point E)

During a cruise of R/V *Seikai* (Japan Atomic Energy Agency) in July 2011 in the shallow region (bottom depth < 50 m) of the coastal area of Ibaraki Prefecture, a sediment sample was collected with a Smith–McIntyre sampler at point E, and the upper layer (0–3 cm) was separated from the rest of the grab sample on board. The sample consisted of very fine sand/fine sand (0.0625–0.25 mm; Wentworth scale). After transfer to a laboratory on land, the sample was dried at 105 °C and crushed, and the coarse fraction was removed by using a 2-mm sieve. The sieved sample was transferred to a plastic container, and gamma-rays specific to ^134^Cs (604.7 and 795.9 keV) and ^137^Cs (661.7 keV) were measured by using a coaxial Ge detector (ORTEC GEM20P4, resolution 1.7 keV/1.33 meV, relative efficiency 29%–31%). This measurement has been described in detail by Otosaka and Kobayashi^41^ and Otosaka and Kato^6^.

#### (5) Suspended particles collected by water filtration (point F)

At point F, 60 L of estuarine water was filtered through a sieve with 64-µm pore size in November 2012. The water was then filtered in situ through 3-µm and 0.45-µm pore size membrane filters by using an ADVANTEC pressurised filtering system (DV-10 and KS-142-UH, Advantec, Tokyo, Japan). The filters were dried at 60 °C for 12 h, and then the suspended particles (3–63 µm) on the filters were placed into a custom plastic bag and sealed for later gamma-ray measurement. The sample was measured for ^137^Cs (661.7 keV) by using a planar-type Ge detector (GC4018/7915-30/ULB-GC, CANBERRA). This measurement is explained in detail by Sakaguchi et al*.*^42,43^.

### Isolation of RPs from bulk samples

The spatial distribution of radioactivity, particularly RCs, in each bulk sample was measured by autoradiography with an IP (BAS-MS 2040, 130 Fujifilm Corp., Japan) and an IP reader (FLA-9000, Fujifilm Corp., Japan). Three CsMPs isolated from terrestrial samples^17^ with known activity (~ 1, ~ 4, and ~ 20 Bq of ^137^Cs) were measured along with the samples as calibration standards for calculating ^137^Cs activity at each hot spot. High radioactive spots in the samples identified on the images by the IP reader were considered to be CsMPs. Particles with relatively high radioactivity (RPs) were isolated from the samples by the wet separation method, which is an efficient method of isolating RPs^16,17,21^. After isolation, each RP in the separation water was carefully dropped onto a Kapton tape and air-dried for subsequent analysis.

### Measurement of ^134^Cs and ^137^Cs activities in the isolated RPs

The ^134^Cs (604.7 keV) and ^137^Cs (661.7 keV) activities of the isolated RPs were determined by gamma-ray spectrometry with a HPGe (GX4018, CANBERRA Industries 142 Inc., USA) to determine the FDNPP unit from which the CsMPs originated. Radioactivity standard solutions for ^134^Cs (0.182 Bq as of 25 November, 2016, Japan Radioisotope Association, CZ-010) and ^137^Cs (1.40 Bq as of 25 November, 2016, Japan Radioisotope Association, CS-005) dispersed on a filter over an area of 1 mm^2^ were used for calibration of the gamma-ray spectrometer. These standard radioactivity solutions were calibrated by the Japan Calibration Service System (JCSS; http://www.nite.go.jp/en/iajapan/jcss/index.html).

### SEM-EDS analysis of the isolated RPs

SEM (TM3030Plus, Hitachi, Japan) with EDS (AZtecOne, Oxford Instruments, UK) analysis was performed to observe the shape and elemental composition of RPs. Elemental maps of some RPs were also obtained by EDS measurement.

### Calculation of the ratio of ^137^Cs activity in RPs to total ^137^Cs in the bulk sample

To assess the influence of RPs on *K*_d_ and CF values, we calculated the ratio of ^137^Cs activity in RPs to the total ^137^Cs in the bulk samples, R(RPs):

$${\text{R}}\;({\text{RPs}})\;(\% ) \, = {\text{ A}}\;({\text{RPs}}){\text{ / A}}\;({\text{bulk}}) \, \times \, 100$$

where A(RPs) is the ^137^Cs activity in RPs and A(bulk) is the total ^137^Cs activity in the bulk sample containing the RP. A(RPs) is the sum of ^137^Cs activity of the isolated RPs measured by HPGe detector and the ^137^Cs activity of radioactive spots (> 0.1 Bq of ^137^Cs) calculated from the PSL of IP images. A(bulk) was measured by a HPGe detector.

## Supplementary Information

Supplementary Information.
