# Supplementary material for: First isolation and analysis of caesium-bearing microparticles from marine samples in the Pacific coastal area near Fukushima Prefecture
Source: Sci Rep. 2021 Mar 11;11:5664. doi: 10.1038/s41598-021-85085-w (PMC7952385; doi:10.1038/s41598-021-85085-w)
Supplement: Supplementary file 1 — Supplementary Information. [file 41598_2021_85085_MOESM1_ESM.pdf]

First isolation and analysis of caesium-bearing microparticles from marine samples in the Pacific coastal area near Fukushima Prefecture

Authors
Hikaru Miura, Takashi Ishimaru, Yukari Ito, Yuichi Kurihara, Shigeyoshi Otosaka, Aya Sakaguchi, Kazuhiro Misumi, Daisuke Tsumune, Atsushi Kubo, Shogo Higaki, Jota Kanda, and Yoshio Takahashi

Table S1 Sampling information for the bulk samples.

Sampling point	Sample name	Date	Latitude	Longitude	Sample type and sampling method	Water depth (m)	Sampling depth (m)	Filtered water volume (L)	Fraction	Total weight (g)
A	P-43-I01	2015/7/29	37°13′58″ N	141°07′27″ E	Suspended particles collected by water filtration	63	53	2583	> 0.45 μm	0.301
										0.361
B	Hisanohama	2013/12/16	37°11′18″ N	141°01′15″ E	Suspended particles collected by water filtration	15	1.5 m from the bottom	630	> 0.45 μm	1.64
C	UT06	2011/7/2	37°01′05″ N	140°59′35″ E	Zooplankton collected by plankton net	40	1	119300	> 330 μm	0.130
D	GST#1	2014/10/17	37°22′41″ N	141°03′42″ E	Sinking particles collected by sediment trap	20	17	–	–	6.94
	GST#2	2014/10/26								11.8
	GST#3	2014/11/4								7.14
	GST#4	2014/11/13								71.7
	GST#5	2014/11/22								25.5
	GST#6	2014/12/1								31.5
	GST#7	2014/12/10								27.0
E	4UB06	2011/7/20	36°46′06″ N	140°47′18″ E	Marine sediment collected by grab sampler	28	28	–	< 2 mm	7.05
F	Watari	2012/11/30	38°03′10″ N	140°54′51″ E	Suspended particles collected by water filtration	–	1	60	3–63 μm	2.04

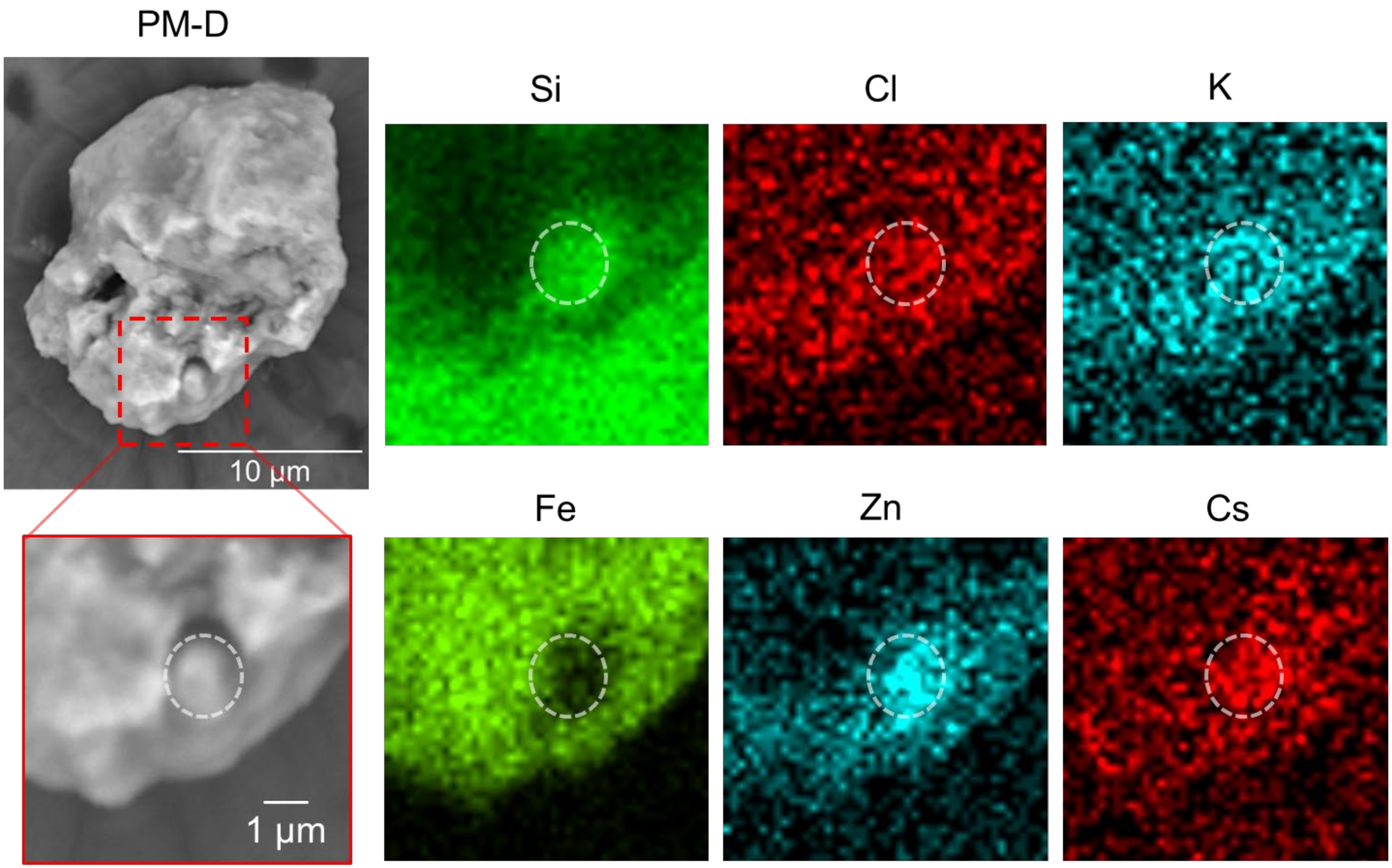

Fig. S1. Elemental maps of the PM-D. Cs was detected in the area within the white dashed circle.

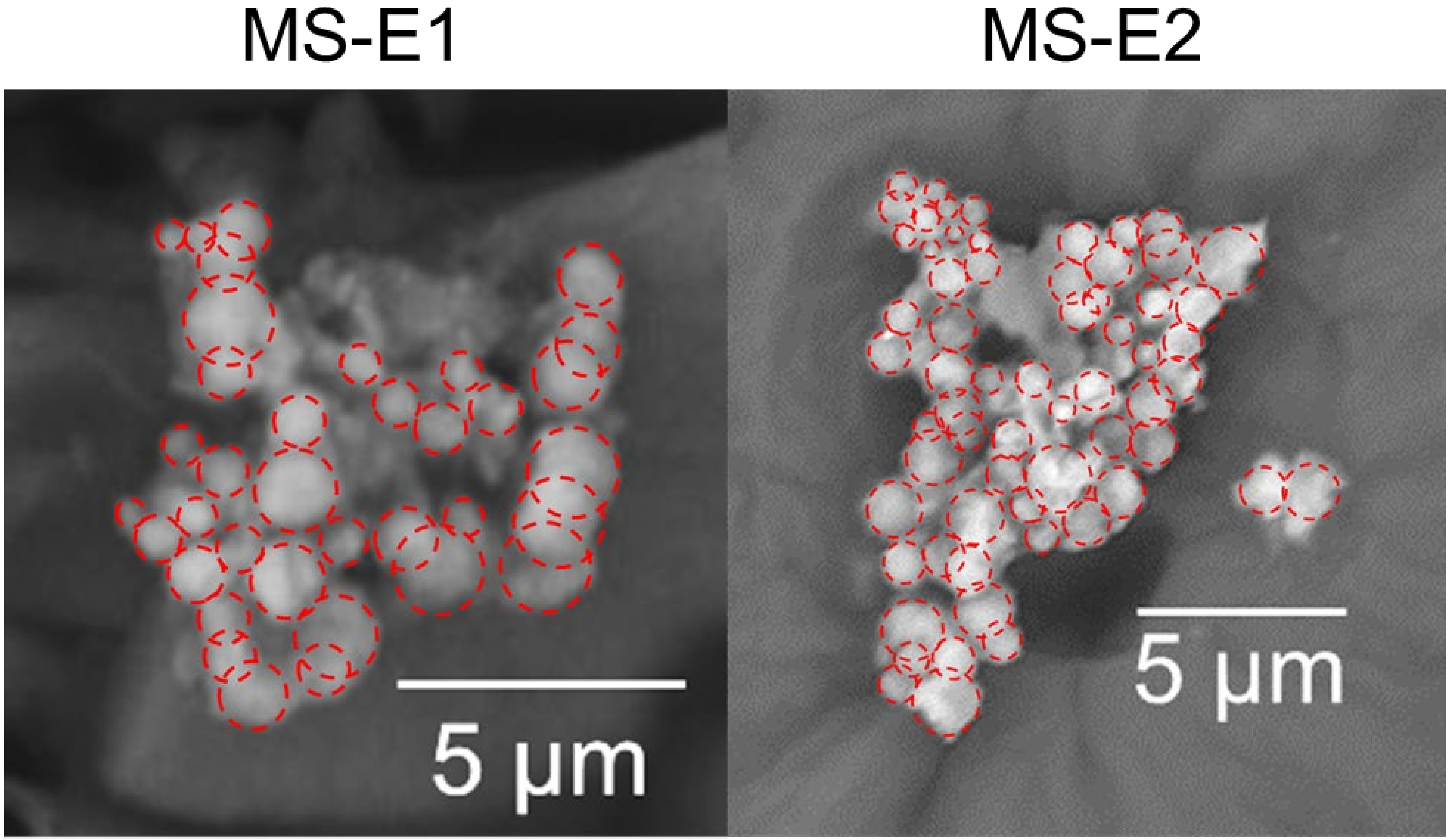

Fig. S2. Approximation of the volumes of MS-E1 and -E2 using spheres (red circles).

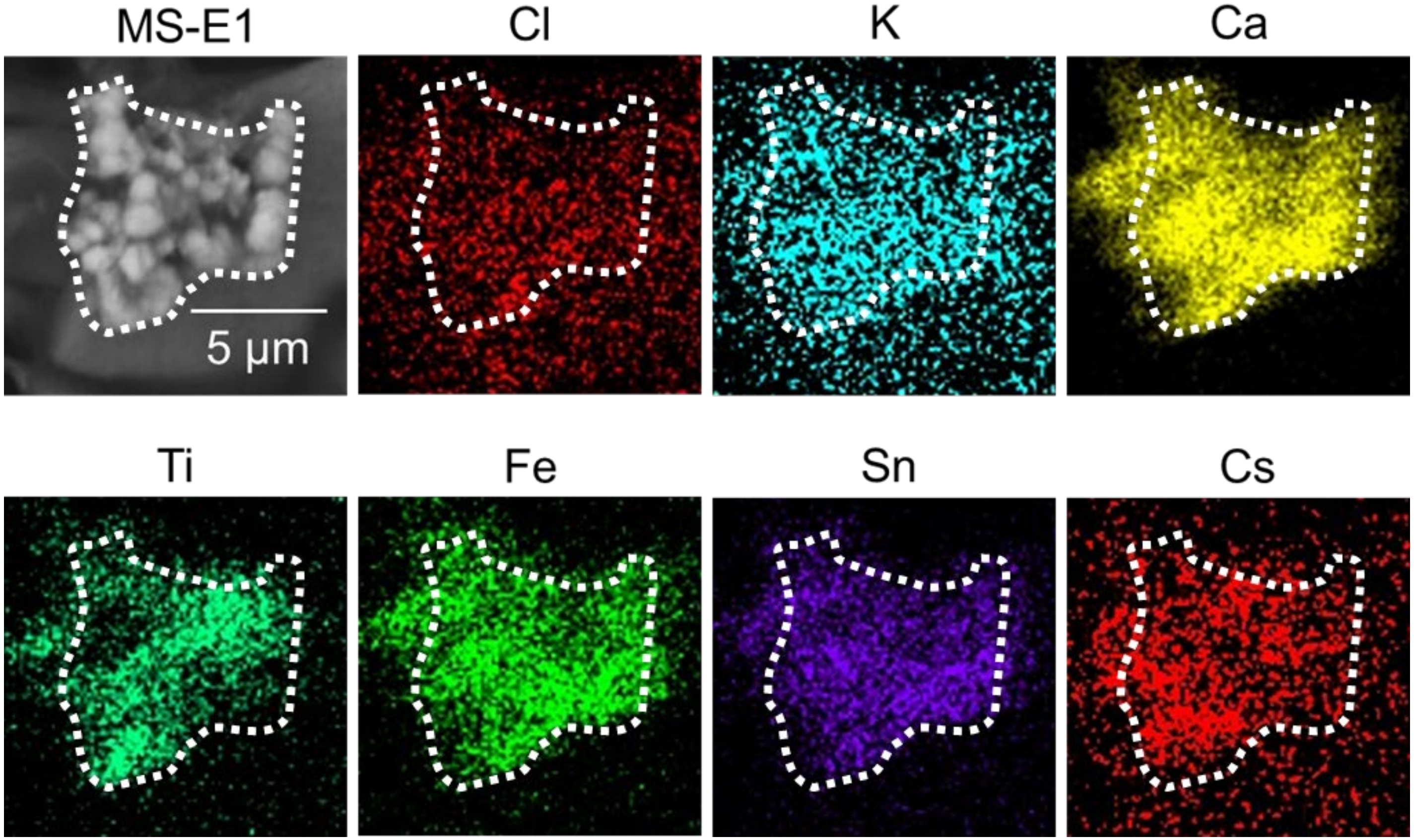

Fig. S3. Elemental map of MS-E1.

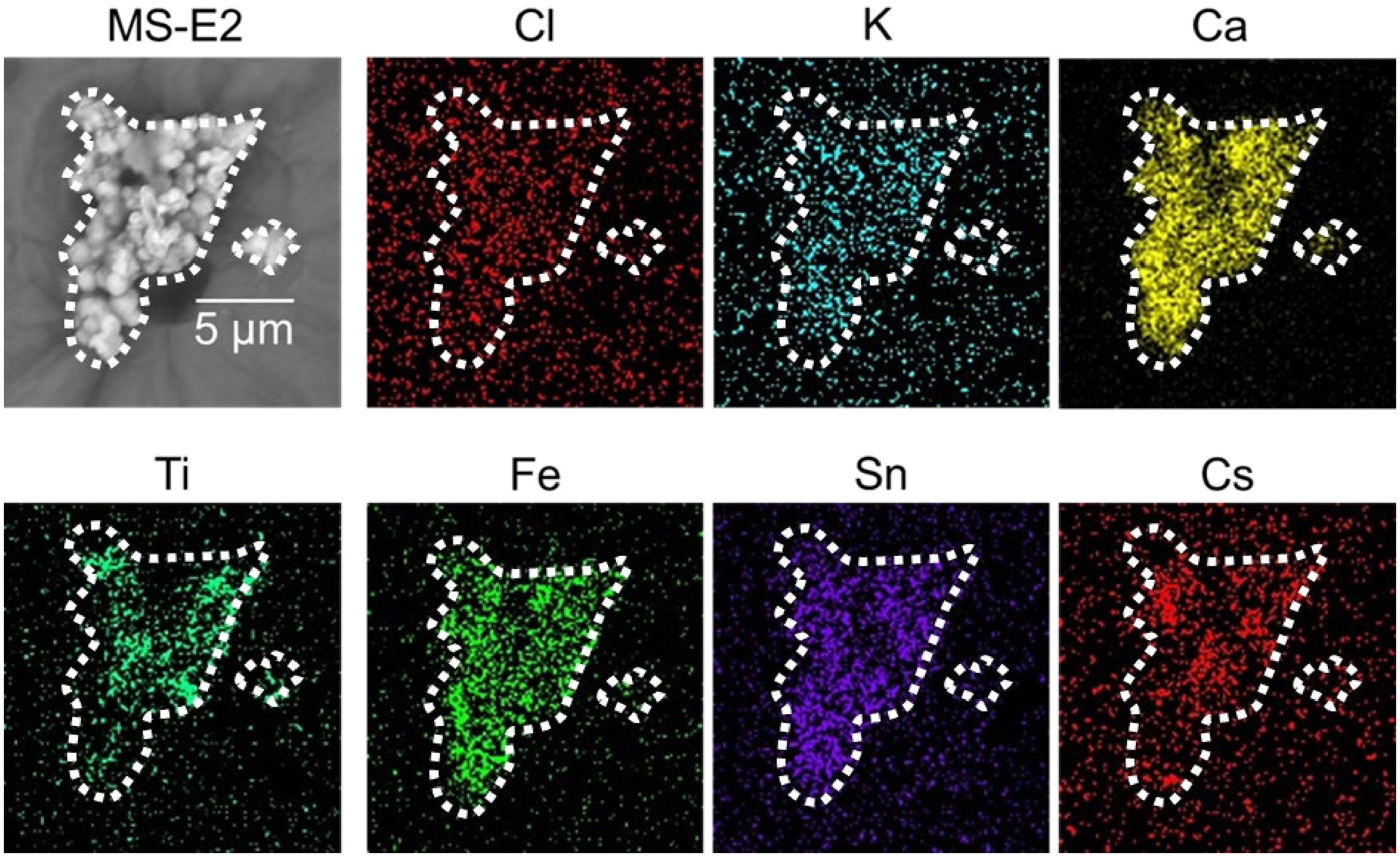

Fig. S4. Elemental map of MS-E2.
